# Nutritional Status of Breastfeeding Mothers and Impact of Diet and Dietary Supplementation: A Narrative Review

**DOI:** 10.3390/nu16020301

**Published:** 2024-01-19

**Authors:** Alejandra Carretero-Krug, Ana Montero-Bravo, Carmen Morais-Moreno, Ana M. Puga, Mª de Lourdes Samaniego-Vaesken, Teresa Partearroyo, Gregorio Varela-Moreiras

**Affiliations:** 1 Grupo USP-CEU de Excelencia “Nutrición para la vida (Nutrition for Life)”, Ref: E02/0720, Departamento de Ciencias Farmacéuticas y de la Salud, Facultad de Farmacia, Universidad San Pablo-CEU, CEU Universities, Urbanización Montepríncipe, 28660 Boadilla del Monte, Spain; alejandra.carreterokrug@ceu.es (A.C.-K.); amontero.fcex@ceu.es (A.M.-B.); carmen.moraismoreno@ceu.es (C.M.-M.); anamaria.pugagimenezazca@ceu.es (A.M.P.); l.samaniego@ceu.es (M.d.L.S.-V.); t.partearroyo@ceu.es (T.P.); 2 Instituto CEU Alimentación y Sociedad, Facultad de Farmacia, Universidad San Pablo-CEU, CEU Universities, Urbanización Montepríncipe, 28660 Boadilla del Monte, Spain

**Keywords:** breastfeeding, lactating, fatty acids, vitamins, minerals, supplementation

## Abstract

Adequate nutrition during breastfeeding is crucial for ensuring the good health of mothers and babies. Despite the high energy and nutrient demands of breastfeeding, lactating women are often vulnerable from a nutritional perspective. The nutritional focus during breastfeeding tends to be on the newborn, often neglecting the mother’s diet. Therefore, in the present narrative review, nutrient intakes were compared with the dietary reference values (DRVs) proposed by the European Food Safety Authority (EFSA) as well as by the World Health Organization/Food and Agriculture Organization (WHO/FAO). In the diets of lactating mothers, dietary inadequacies were observed in the intake of some vitamins, such as folic acid, vitamin B12, vitamin A, and vitamin D, and in the intake of certain minerals like calcium, iron, and iodine; polyunsaturated omega-3 fatty acid deficiencies, primarily in eicosapentaenoic acid and docosahexaenoic acid, were also observed. On the other hand, the debate on the necessity of supplementation during lactation continues; the need for nutritional supplementation during lactation depends on many factors, such us mothers’ eating habits. There seems to be a positive association between nutritional supplementation of the lactating mother and the concentration of certain nutrients in human milk. The present narrative review provides an update on the nutritional status (fatty acids and micronutrients) of breastfeeding mothers and the impact of diet and dietary supplementation on human milk composition.

## 1. Introduction

Adequate nutrition during breastfeeding is of vital importance in ensuring good health in both the mother and the baby [1,2]. Furthermore, breastfeeding provides personalized and unique nutrition and is associated with long-term benefits for both the child’s and the mother’s health [3,4]. 

Breastfeeding is a period of high energy and critical nutrient demands. Therefore, it is a stage where women are particularly vulnerable from a nutritional perspective. It has been observed that even if lactating mothers do not have an adequate nutritional status, they can produce sufficient and high-quality human milk, although this may result in a depletion of maternal reserves. In fact, there are many factors that affect human milk composition. This is mainly influenced by the mother’s dietary intake, her body composition, and maternal fat stores [2,5]. Some studies have additionally highlighted how geographical, cultural, and socioeconomic factors may also affect the nutritional quality of human milk [6]. These factors are quite evident when comparing human milk produced by breastfeeding mothers in developing countries and that of those living in industrialized areas. When a lactating woman resides in a disadvantaged socioeconomic environment, nutritional deficiencies are frequently observed, which can negatively impact her health [7]. However, it is important to note that, in developed countries, even though women usually have access to an adequate amount and varieties of food, the nutritional status of breastfeeding mothers is not always ideal. Moreover, unbalanced food intake and inadequate nutrient density can be observed, leading to several problems (anemia, excess weight, cardiovascular diseases) [8,9,10]. Additionally, during the breastfeeding period, attention is focused primarily on the newborn, and surprisingly not much consideration is given to the diet of the lactating mother [2].

Given the key role of the maternal diet during lactation, there is still an ongoing debate on the need for nutritional supplementation during breastfeeding; it seems that this depends on several factors, such as socioeconomic status or the dietary habits of the mother. For example, in low socioeconomic settings, supplementation has been shown to be an effective strategy to address nutritional deficiencies [11]. In developed countries, however, supplementation has primarily focused on long-chain polyunsaturated fatty acids (PUFAs)—mainly *n*-3 fatty acids—which have been linked to improved neonatal health as indicated by Valentine et al. [12]. They hypothesized that DHA supplementation in mothers decreases inflammatory markers in infants.

Therefore, this narrative review was conducted to provide an update on the available literature regarding the nutritional status (fatty acids and micronutrients) of breastfeeding mothers, and the influence that diet and dietary supplementation have on the composition of human milk. Therefore, nutrient intakes were compared with the dietary reference values (DRVs) proposed by the European Food Safety Authority (EFSA) as well as by the World Health Organization/Food and Agriculture Organization (WHO/FAO).

## 2. Materials and Methods

### 2.1. Search Methods

A literature search was carried out using the following databases: MEDLINE, PubMed, and Scopus, using the following search terms: “Breastfeeding”; “lactating”; “lactation”; “nutrition”; “micronutrients”; “vitamins”; “minerals”; “fatty acids”; “omega 3”; “supplementation”; “supplements”; AND “dietary goals” to identify relevant studies published up to April 2023. Sources published within the past 10 years were preferentially selected. Randomized controlled trials, cohort studies, systematic reviews, and meta-analyses were included. Only articles conducted in humans and published either in English or Spanish were considered. 

### 2.2. Selection Criteria and Eligibility

Eligible populations included breastfeeding women of all age groups. Articles were eligible if they included nutritional status and/or supplementation with vitamins, minerals, or fatty acids. 

## 3. Nutritional Status of the Breastfeeding Mother

### 3.1. Fatty Acid Intake during Lactation

Arachidonic acid (AA, C20:4 *n*-6) and docosahexaenoic acid (DHA, C22:6 *n*-3) are essential long-chain PUFAs that play an important role in several metabolic and physiological processes during embryonic and fetal development, as well as in the early years of life [13]. The highest AA and DHA concentrations are found in the nervous system, particularly in the brain, and the retina, specifically in the phospholipids of cell membranes. AA and DHA actively participate in brain development, neuronal differentiation, and overall energy and metabolic status [14]. AA is derived from the precursor linoleic acid (LA, C18:2 *n*-6) and DHA is derived from the precursor alpha-linolenic acid (ALA, C18:3 *n*-3) [15]. They have opposing functions in the human body. Despite the key functions of AA, no regulatory agencies have suggested a requirement for this *n*-6 fatty acid. While *n*-6 fatty acids serve as precursors for eicosanoids with inflammatory consequences, *n*-3 fatty acids are precursors for eicosanoids with anti-inflammatory effects. In fact, excessive *n*-6 fatty acid intake above recommended levels, may affect *n*-3 fatty acid availability. High *n*-6/*n*-3 ratios (15/1) seem to favour inflammation and increase proneness to chronic diseases (cardiovascular, inflammatory, or autoimmune) [16].

Table 1 includes the nutritional goals recommended by international organizations regarding fat intake in healthy adult populations and evidence of compliance with nutritional recommendations.

In Western countries, DHA intake from fish is scarce compared to that of LA and AA (from vegetable oils and eggs and meat, respectively) [30]. In addition to the greater *n*-6 fatty acid dietary intake, AA synthesis from LA is more efficient than DHA synthesis from ALA [31]. It is important to emphasize the very limited metabolic conversion of dietary ALA into DHA, as this conversion may not be substantial enough to promote optimal health in a significant portion of the population, especially among those who do not include fish and shellfish in their diets [32]. Several studies have established the relationship between the fatty acid composition of human milk and the maternal dietary intake of fatty acids during pregnancy and lactation [33]. Tissue fatty acid levels during pregnancy and lactation are directly related to a woman’s diet, capacity for storage, and their metabolic use of fatty acids (including synthesis, oxidation, or transport) [34,35]. Therefore, both diet and fatty acid metabolism may determine the long-chain fatty acid concentration in human milk [36]. The availability of long-chain fatty acids for the infant is dependent on the transfer of these nutrients from the mother to her offspring, first through the placenta and then through breastfeeding. As for AA and DHA, their availability will depend on food consumption and/or the mother’s capacity to synthesize these fatty acids from their metabolic precursors [37]. Fatty acids from the mother’s diet are transferred to human milk (within six hours) and can remain in human milk for days after initial intake [38]. 

Some authors have highlighted that, in industrialized countries, the lipid profiles of the diets of lactating mothers are inadequate. In the MEDIDIET study [1], conducted in a group of 300 Italian mothers, an average intake of saturated fatty acids (SFAs) of 23.5 g/day (10.8%) was observed, which is significantly lower than that observed in other studies in Mediterranean countries [20,21]. Likewise, the intake of monounsaturated fatty acids (MUFAs) was 29.4 g/day (13.5%), which is also lower than reported in other studies. The diets of women in the MEDIDIET study also exhibited a low intake (8.1 g/day; 3.7%) of PUFA, resulting in higher values for *n*-6 fatty acids (6.7 g/day and 3.1% of total energy intake) and LA (6.4 g/day) than seen in other studies in Mediterranean countries [20,21]. The intake of ALA was 1.1 g/day. 

Long-chain *n*-3 fatty acids, eicosapentaenoic acid (EPA), and DHA contribute to the visual and cognitive development of the baby during pregnancy and the child during lactation [39]. The infant’s intake of *n*-3 fatty acids depends directly on their transfer through human milk [40]. Moreover, dietary DHA has a strong dose-dependent effect on DHA concentration in breast milk [41].

Furthermore, the MEDIDIET study [1] also showed values of EPA and DHA—intakes of 0.13 g/day (EPA) and 0.17 g/day (DHA)—close to those recommended by the WHO (adequate intake of EPA + DHA = 0.3 g/day). The high concentrations of MUFA (in terms of total fat percentage) could be attributed to a high consumption of olive oil (typical of the Mediterranean diet). 

In a systematic review by Di Masso et al. (2021) (MEDIDIET Study) [1], it was observed that the intake of SFA (expressed in g/day) ranged from 13.4 g/day for lactating Chinese women who participated in the study by Xiang et al. (2005) [22] to 45.0 g/day for Icelandic mothers (*n* = 77) in the study by Olafsdottir et al. (2006) [23]. This study also found that the group of Icelandic mothers who regularly consumed cod liver oil had a significantly higher total intake of PUFA (14 g/day; 5% TE) compared to those who did not consume it (9 g/day; 3.9%). Specifically, mothers who consumed cod liver oil showed greater concentrations of EPA and DHA in their milk; however, their intake of MUFA (11.9%) was below that of the WHO/FAO recommendations [18].

On the other hand, Butts et al. (2018) [24], in a study conducted on 78 mothers (aged 19–42) of Asian, Māori (Pacific Islander), or European origin residing in New Zealand, found that the intake of SFA was high in all three groups studied (11.9%, 15%, and 15.2% of total energy consumed, respectively). Similarly, Mojska et al. (2003) [25] assessed the diets of 69 Polish lactating mothers and found that SFA intake (12.6%) was also above that of the WHO recommendations (<10%), MUFA intake was slightly below that of recommendations (14.5%), while PUFA intake (5.7%) met that of the recommendations. An imbalanced lipid profile was also observed in another study by Krešić et al. (2012) [20] conducted in 83 Croatian lactating mothers, where a very high intake of SFA (14%) was noted; although, in this case, the intakes of MUFA and PUFA met that of the recommendations (15.2% and 6.7%, respectively).

Meanwhile, Kim et al. (2017) [28] assessed the consumption of PUFA in a total of 238 lactating mothers from South Korea and observed an intake of *n*-6 fatty acids of 9.9 g/day and a very low intake of *n*-3 fatty acids (1.2 g/day). Of the *n*-3 fatty acids, 0.14 g/day corresponded to DHA and 0.07 g/day corresponded to EPA. In a study by Xiang et al. (2005) [22], an intake of *n*-6 fatty acids of 10.1 g/day was observed in Swedish mothers and 14.1 g/day in Chinese mothers. The intake of *n*-3 fatty acids, however, was very similar in the two population groups: 1.8 g/day (0.01 g/day of DHA) in the group of Chinese lactating mothers and 1.7 g/day in the group of Swedish mothers. In this case, they consumed 0.09 g/day of EPA and 0.12 g/day of DHA, which represented a higher DHA intake value than that of the group of Chinese lactating mothers.

Aumeistere et al. (2019) [19] assessed the intake of 61 Latvian women aged between 23 and 39 years and observed that their total and saturated fat intakes surpassed those of the recommendations. The intakes of LA and ALA were adequate, but the intake of DHA was significantly lower than that recommended. The fatty acid profile of human milk was influenced by the mother’s recent dietary intake. Moreover, significant positive correlations were observed between maternal intake of linoleic acid, ALA, DHA, MUFA, and PUFA, and their contents in human milk. 

In a study by Barrera et al. (2018) [26] conducted on 50 Chilean lactating mothers aged 20–33 years (all of whom belonged to low–middle socioeconomic strata), a high intake of SFA and acceptable total MUFA intake were observed. However, they had a very low intake of total *n*-3 fatty acids, EPA, and DHA (0.02 g/day and 0.04 g/day, respectively) during the first month of lactation, which decreased by another 50% in the sixth month of lactation. This could be attributed to the low consumption of fish and seafood and the high consumption of soybean or sunflower oil.

On the other hand, Sabel et al. (2009) [27] studied the relationship between the fatty acid statuses of a group of Swedish lactating mothers (29 mothers of singletons and 11 mothers of twins) and their premature infants (24–36 weeks’ gestation, without intensive care requirements). Omega-3 intake was assessed, providing 0.7% TE—a value lower than that of recommendations (1% TE). Previous studies had indicated that low omega-3 intake could contribute to premature birth [29], which was supported by the results of this study. The average ALA intake was 1.47 g/day, and the EPA + DHA intake was 0.20 g/day. Finally, in this study, 90% of the sample had essential fatty acid intakes below recommended levels. 

It should be noted that it is not easy to change the dietary habits of women during breastfeeding [42,43]. In this population group, there is often an insufficient intake of omega-3 fatty acids due to low consumption of fatty fish, as there is currently great concern about the contamination of marine species with heavy metals [44]. Aumeistere et al. (2018) [45] studied the relationship between fish consumption and DHA levels in human milk, and observed a positive correlation between fish, butter, and avocado consumption with DHA levels in human milk. Furthermore, they found that mothers who eat fish—at least two servings per week—had significantly higher concentrations of DHA in human milk vs. those who did not. This relationship has also been observed in other studies [46,47,48,49]. Additionally, Quin and Kuawa (2012) [50] concluded that the addition of one serving of fish per week resulted in a 0.014% increase in DHA in human milk. However, it should be emphasized that other foods, such as eggs and meat, are also sources of DHA [46,51]. To address possible deficiencies in the intake of this fatty acid, Echevarria et al. (2017) [52], in a review, recommend dietary supplements containing omega-3 fatty acids (specifically, DHA) or foods enriched or fortified with this fatty acid. Sherry et al. (2015) [53] showed that after 6 weeks of supplementation with a low or high dose of DHA, absolute human milk DHA was significantly greater than that of placebo.

### 3.2. Dietary Micronutrient Intake of the Breastfeeding Mother

Adequate micronutrient intake is particularly important during lactation [54,55]. During this stage, micronutrients play a very crucial role in the neural development of the newborn, metabolic processes, tissue and muscle synthesis, oxygen transport, and DNA synthesis, among other processes. Additionally, some show an antioxidant effect and are essential for the proper functioning of the newborn’s immune system [56].

#### 3.2.1. Vitamins

The nutritional recommendations for vitamins for lactating mothers are shown in Table 2 and Table 3, categorized into fat-soluble and water-soluble vitamins. Also, evidence of compliance with nutritional recommendations are presented in these tables. 

Vitamin intake in lactating mothers has been assessed by many studies. Moran et al. (2013), in a study [63] conducted on 310 women during pregnancy and lactation (4 months postpartum), observed that those women had deficient vitamin A (1618.3 µg), vitamin B1 (2.9 mg), vitamin B2 (3.5 mg), niacin (29.6 mg), vitamin B6 (4.3 mg), vitamin C (181.9 mg), and folate (893 µg) intakes. Daniels et al. (2019) [61] also conducted a study on 113 Indonesian women exclusively breastfeeding their children (average age 25.8 years). In this group of lactating mothers, insufficient intake of vitamin A (501 µg RAE), niacin (12.8 mg), vitamin B6 (1.3 mg), and vitamin B12 (2.5 mg) was observed. Furthermore, the mothers’ habitual daily intakes of vitamin A, niacin, and riboflavin (1.7 mg) were positively associated with the concentrations of these micronutrients in human milk. Likewise, Copp et al. (2018) [62] analyzed the diets of 16 lactating American women, finding that their mean intake met the U.S. Recommended Dietary Allowances (RDAs) for vitamins B1 (1.9 mg), B2 (2.2 mg), B6 (1.9 mg), and B12 (5.0 µg). However, the average intake of vitamin A (753.1 µg RAE), vitamin D (6.6 µg), and choline (317.1 mg) did not meet the recommendations, as they only covered 58% of the recommended intake for vitamin A, 44% of that for vitamin D, and 58% of that for choline. On the other hand, the ATLAS study (2021) [64], conducted on lactating mothers from various European countries, revealed that the consumption of vitamin A (819 µg), vitamin C (120.5 mg), and vitamin D (5.3 µg) was considerably below the recommendations established by the EFSA. However, the recommendations for thiamine (1.6 mg) and niacin (21.3 mg) were met. Similar results were found in a study by Gila-Diaz et al. (2021) [2], conducted on 30 Spanish lactating women, where inadequate intakes of vitamin D, vitamin A, and folic acid were observed, while the intakes of vitamins B1, B2, B3, B6, and B12 were adequate (above those of recommendations). This could be related to the fact that the vast majority of lactating mothers consumed a sufficient number of fruits and vegetables, demonstrated high adherence to the Mediterranean healthy food pyramid, and a high percentage of them took vitamin and mineral dietary supplements [2]. However, as mentioned, vitamin A, vitamin D, and folic acid intakes fell below recommended levels. Maternal intake was assessed on day 7 and day 28 postpartum, and no significant differences were observed at these two time points. Moreover, only vitamin A (1046 vs. 1381 µg) and B9 (306 vs. 498 µg) intakes were significantly lower on day 7, while vitamin E (10.4 vs. 17.9 mg) intake was significantly higher on day 28 postpartum. In the case of vitamin E, it was observed that its levels comfortably met those of the recommendations for this population group (it is worth noting that vitamin E is especially important in the initial periods of life, as it acts as an antioxidant defence against oxygen-induced toxicity in the extrauterine environment). 

However, lactating mothers in the already mentioned MEDIDIET study [1] generally had vitamin intakes that either met or came close to recommendations for this population group. In the case of vitamin D, though, their intakes were well below recommended levels (2.6 μg/day vs. the adequate intake (AI) of 15 μg/day). They showed an intake of 1143 μg/day for vitamin A, 1.0 mg/day for thiamine (which corresponds to a very low intake compared to that observed in other studies of lactating mothers), 1.6 mg/day for riboflavin, 18.9 mg/day for niacin equivalents, and 2.2 mg/day for vitamin B6. The folate intake of 330 μg/day was slightly below recommended levels. However, intake of vitamin C met that of recommendations (184 mg/day). 

Similar results were obtained by Bzikowska-Jura et al. (2021) [59], who studied the diets of 40 Polish lactating women at three different time points: 1, 3 and 6 months of lactation. They observed that there were no significant differences in vitamin intake between the three time points in the study. They also observed adequate intakes of vitamin A (1049.3–1390 µg/day), vitamin E (10.4–11.9 mg/day), vitamin B1 (1.2–1.4 mg/day), vitamin B2 (1.6–1.8 mg/day), vitamin B6 (1.9–4.8 mg/day), vitamin C (127.8–163 mg/day), and vitamin B12 (3.2–4.04 µg/day). However, most of the women in the study consumed less than the recommended intakes of folic acid (310–436.5 µg/day) and vitamin D (2.7–3.2 µg/day).

It is worth noting the generalized low intake of vitamin D that women show during breastfeeding, which is well below the recommended values. Vitamin D is essential for guaranteeing calcium homeostasis and bone health. In addition, evidence suggest an important role for vitamin D in innate and autoimmune functions [66]. Vitamin D deficiency is harmful to both the mother and her children, leading to an increased risk of osteomalacia in adults and rickets and growth delay in infants [67,68]. Additionally, it has been observed that a lack of vitamin D is related to a higher risk of autoimmune diseases in adults and children [68], and respiratory tract infections in children [67]. Vitamin D deficiency is considered a public health concern worldwide, even in countries with plenty of sunlight [69].

Vitamin D deficiency is widespread in Europe [69], and breastfeeding represents a critical period in a woman’s vitamin D status throughout her life. A study conducted in Germany showed that 49% of pregnant women during summertime and 98% of pregnant women in winter displayed inadequate levels of vitamin D before birth [65], indicating that the breastfeeding period began in a vitamin D-deficient environment. Gellert et al. (2017) [70] conducted a study in German lactating women to assess their vitamin D statuses and compared them to those of non-pregnant, non-lactating women without dietary supplementation. This study revealed an inadequate vitamin D status in lactating women, as well as in a control group of non-pregnant, non-lactating women. They reported that the prevalence of deficient (<25 nmol/L) and insufficient (<50 nmol/L) vitamin D was significantly higher in lactating women (26.6% and 75.8%, respectively) than in the control group (12.9% and 58.9%, respectively). Additionally, they found that lactating women had a higher probability of vitamin D deficiency (<25.0 nmol/L) than the control group, which might be partly explained by the loss of vitamin D through human milk [71]. Furthermore, this study showed higher vitamin D concentrations in lactating women during the summer and autumn compared to the spring and winter. However, a study conducted in Sweden [60] demonstrated insufficient vitamin D levels (<50 nmol/L) in lactating women (at 2 weeks, 4 months, and 12 months postpartum) in the winter months (November–April) and a prevalence of 15% in the summer months. No significant variations in vitamin D levels were observed throughout the postpartum period (2 weeks, 4 months, and 12 months), considering that 18% of the study sample received vitamin D supplementation. Another study conducted globally [72] in the Chinese, American, and Mexican populations found that 43% of lactating women (4 weeks postpartum) had insufficient vitamin D levels (<50 nmol/L), with vitamin D supplementation rates varying from 22% to 94% in these countries. 

Other studies have also observed a higher prevalence of vitamin D deficiency. A total of 61% of lactating women in the United Arab Emirates [73], 48% of lactating mothers in India [74], and 46% of lactating mothers in Turkey [75] had severe vitamin D deficiency (<25.0 nmol/L). Dawodu et al. (2015) [72] studied the prevalence of vitamin D deficiency in a cohort of lactating mothers from China, EEUU (Ohio), and Mexico. They observed a deficiency (<50 nmol/L in blood) rate of 62% in the Mexican sample, followed by 52% in Chinese women, and 17% in Ohio women. In this study, the low prevalence of vitamin D deficiency in the Ohio sample was due to high sun exposure and substantial supplementation in this group (87% declared vitamin D supplementation). Altogether, the prevention of vitamin D deficiency, as well as the screening of breastfeeding women for the identification of vitamin D deficiency and to allow prompt treatment, seems to be crucial during breastfeeding.

To summarize, low serum vitamin D concentrations in lactating women may be related to a low consumption of vitamin D-containing foods, as well as limited sun exposure [76]. Vitamin D transfers from maternal serum to human milk [70,77], suggesting that the babies of mothers with dietary vitamin D inadequacies may also display a lack of this vitamin. Although mothers are usually supplemented with this vitamin during pregnancy, these results indicate that supplementation should continue during lactation to increase the concentration of this vitamin in human milk [78]. 

#### 3.2.2. Minerals

The nutritional recommendations for minerals for lactating mothers are shown in Table 4; evidence of compliance with nutritional recommendations is also shown.

Data concerning the mineral intakes of calcium (mg/day), phosphorus (mg/day), potassium (mg/day), iron (mg/day), zinc (mg/day), iodine (µg/day), and magnesium (mg/day) are analyzed and discussed below. 

Regarding calcium intake, Gila-Díaz et al. (2021) [2], in a study conducted on breastfeeding mothers (first month postpartum), which analyzed their diets on the 7th and 28th postpartum days, observed calcium intakes of 986 mg/day on day 7 and 1094 mg/day on day 28 of lactation, both of which were in accordance with the reference intakes established by the EFSA (1000 mg/day). Similar intakes were observed in other studies conducted on breastfeeding mothers in Spain (1102 mg/day) [81], Croatia [20] (994 mg/day), and México [80] (1636 mg/day), as well as in the ATLAS study conducted in several European countries (957 mg/day) [64]. However, calcium intake values below the EFSA’s reference recommendations were observed in the MEDIDIET study [1] (792 mg/día), a study by Bzikowska et al. (2021) during the first month of lactation (746 mg/day) [79], and a study by the same authors in 2018 from the first to the sixth month of lactation (613 to 745 mg/day) [59]. Similar results were also observed by Daniels et al. (2019) [61] in a study conducted on breastfeeding mothers in New Zealand (613 mg/day).

Phosphorus intake was 1358 mg/day in the MEDIDIET study [1], which is very similar to the values obtained by Bzikowska et al. in 2018 (1146–1326 mg/day) [59] and in 2021 (1331 mg/day) [79]. Even higher intake values were reported in studies by Krešić et al. (2012) in Croatian women (1465 mg/day) [20] and Sánchez et al. (2008) in Spanish women (1612 mg/day) [81]. In all cases, the data showed intake significantly higher than the adequate intake (AI) of 550 mg/day proposed by the EFSA.

Potassium intake according to the MEDIDIET study [1] was 3304 mg/day, which is lower than the adequate intake (4000 mg/day) recommended by the EFSA. Similar values were obtained in a study conducted by Gila-Díaz et al. (2021) [2] in the Spanish population, where the potassium intakes of lactating women were 3051–3271 mg/day, as well as in studies conducted on Polish mothers by Bzikowska et al. (2953–3132 mg/day) [59,79] and in the ATLAS study (3043 mg/day) [64]. However, a study conducted by Caire-Juvera et al. (2007) [80] on Mexican lactating mothers showed intake values of 3540 mg/day, which exceeded the reference values. As is known, potassium can influence certain physiological parameters in lactating mothers, such as blood pressure. In fact, it has been demonstrated that a potassium-rich diet has blood pressure-lowering effects [84]. 

Furthermore, the mothers from the MEDIDIET study had an iron intake of 10.8 mg/day, which is similar to what was observed in studies by Krešić et al. [20] (12 mg/day), Sabel et al. (2009) (11 mg/day) [27], Bzikowska et al. (2018, 2021) (11.2–12.8 mg/day) [59,79], and Özden et al. (2015) (9.15 mg/day) [84]. In these studies, the observed intakes were well below the reference intakes proposed by the EFSA (16 mg/day). However, values of intake very close to those of the recommendations were observed by Sánchez et al. (15.2 mg/day) [81], by Caire-Juvera et al. (2007) (16.1 mg/day) [80], in the ATLAS study (2021) (16.4 mg/day) [64], by Daniels et al. (2019) (18.3 mg/day) [61], and by Gila-Díaz et al. (2021) (18.1–27.6 mg/day) [2]. 

The intake of zinc in the MEDIDIET study [1] was 11.4 mg/day, which is close to the recommendations set by the EFSA (12.2 mg/day). Similarly very close to or meeting the recommendations were values reported by Caire-Juvera et al. (2007) [80], who observed an intake of 11.6 mg/day, Daniels et al. (2019) [61], who reported an intake of 12.8 mg/day, and Sabel et al. (2009) [27], who reported an intake of 11.6 mg/day. Below the recommendations were the values obtained in the ATLAS study (2021) (10.2 mg/day) [64], by Bzikowska et al. (2018, 2021) (8.6–10.5 mg/day) [59,79], and by Özden et al. (2015) (8,2 mg/día) [82].

Regarding iodine intake, the recommended adequate intake as per the EFSA is 200 µg/day. This intake was not met in the ATLAS study [64] (156.5 µg/day) nor in a study carried out by Bzikowska et al. (88.7–106.6 µg/day) [59,79]. Threapleton et al. (2021), who conducted a study in the UK [85] on postpartum mothers, observed an iodine intake in the first 30 weeks postpartum of 100 µg/day; when dietary supplements were included, this was 104 (64–152) µg/day. Similar results were also reported by Henjum et al. (2017) [86], where 75% of the breastfeeding mothers from the study did not meet the EFSA’s recommendations. Inadequate iodine intake was also observed by Gila-Diaz et al. (2021) [2] in the first week postpartum (103 µg/day). However, this study showed slightly higher intakes (268 µg/day) on day 28 of lactation, which is above the recommendations. Jin et al. (2022) [87] also observed slightly higher intakes—although close to the reference value—in a study conducted on breastfeeding women from New Zealand, where estimated iodine intake was 212 µg/day. 

Regarding magnesium intake, it was observed that lactating mothers met the recommendations set by the EFSA (300 mg/day) in both the ATLAS study (2021) [64], which showed an intake of 322 mg/day, and in studies conducted by Bzikowska et al. (2018, 2021) [59,79], where magnesium intake was around 320 mg/day. However, in a study by Krešić et al. (2012) [20] in Croatian women, an intake below the recommendations (240 mg/day) was observed.

On the other hand, regarding sodium intake, the EFSA recommends an intake below 2000 mg/day. Adhering to these recommendations, the MEDIDIET study [1] observed an intake of 1903 mg/day in a group of Italian lactating mothers. However, values exceeding the recommendations were observed in studies conducted in lactating mothers from Poland (2314–2666 mg/day) [59,79], the USA (2687.2 mg/day) [83], Mexico (2579 mg/day) [80], and in the ATLAS study performed in a sample from various European countries (2698 mg/day) [64]. 

Regarding the association between maternal dietary mineral intake and human milk concentration, a study conducted on Spanish lactating mothers observed a positive correlation between the zinc content in transitional and mature human milk and the maternal dietary intake of this mineral [88]. Similar results were observed in another observational study conducted on Finnish mothers. In the latter, a positive correlation was also found between the iron content of human milk and maternal dietary intakes [89]. Human milk is the primary source of zinc for newborns, and there are physiological mechanisms through which maternal zinc reserves are mobilized into human milk [90,91] Zinc is involved in various cellular processes, enzymatic functions, and immune responses [92]. In children, it has been associated with growth, the immune system, and cognitive performance [93,94]. Other risk factors of zinc deficiency are diarrheal disease, malabsorption syndromes, and parasitosis [95]. On the other hand, a study [96] conducted on Norwegian lactating mothers showed a relationship between the mother’s iodine intake and urinary iodine levels in their breastfeeding infants, confirming the link between maternal iodine intake, human milk, and therefore the iodine status of breastfeeding infants. This result has also been observed in other studies in Norwegian lactating mothers [86]. Lactating women should consume an adequate amount of iodine to ensure optimal thyroid function for both them and their children [97]. Adequate iodine intake for optimal thyroid function during the first 4–6 months of a child’s life can be guaranteed through the consumption of human milk and, if necessary, infant formula. Iodine concentrations in human milk are directly dependent on maternal iodine intake, making exclusively breastfed infants entirely dependent on their mother’s iodine intake [98]. However, other authors [99] did not find any correlation between the iron, calcium, magnesium, and potassium contents of human milk and maternal dietary intake.

## 4. Effectiveness of Dietary Supplement Consumption during Breastfeeding

Nutritional deficiencies during pregnancy and lactation make women particularly vulnerable. Furthermore, most nutritional demands during lactation are considerably higher than during pregnancy [100]. An intake of vitamins and minerals below recommended levels during lactation can have a negative impact on the health of both the mother and the breastfeeding child. Breastfeeding mothers not only have an increased need for vitamins and minerals due to their physiological state, but they are also more susceptible to health problems caused by these deficiencies [101]. Nutritional deficiencies in this population can vary depending on the country, culture, dietary habits, and the socioeconomic status of the mother [102]. 

As previously stated, human milk composition depends on the mother’s diet. For some micronutrients like thiamine, riboflavin, vitamin B6 and B12, as well as vitamin A, vitamin D, vitamin E, iodine, and selenium (in populations with endemic deficiencies), human milk has been observed as the primary nutritional source for the infant, and the amounts of these micronutrients in human milk can vary greatly based on the mother’s intake. In the cases of calcium, iron, copper, and zinc, maternal status or dietary intake has a scarce effect on these nutrients’ concentrations in human milk. During deficiency, the mother’s body prioritizes the baby’s needs; therefore, these minerals and trace elements continue to be excreted in human milk in sufficient amounts at the expense of maternal stores. Consequently, the infant is comparatively well protected from maternal deficiency, but the mother is at a higher risk of nutritional deficiencies during lactation if her intake does not meet the recommendations [103]. 

In low-income settings, the use of dietary supplements has proven to be effective in addressing nutritional deficiencies [11]. However, in higher-income countries, supplementation during this stage has primarily focused on long-chain PUFAs [104]. 

In most cases, these nutritional deficiencies can be corrected with proper dietary planning and supplement use [54]. Currently, information regarding the role of dietary supplementation during lactation is very limited. Moreover, the most available information largely comes from studies conducted at the beginning of lactation. However, women’s nutritional needs during lactation are directly related to its intensity and duration, which means that assessing the mother’s nutritional status during early lactation (the first few months) may not correspond to her nutritional status after an extended lactation period (>6 months) [105].

### 4.1. Prevalence of Supplementation during Breastfeeding

Here, we present data related to supplementation habits among women during the lactation period. In a study conducted by Cuervo et al. (2014) [106] that studied the nutritional status of Spanish women during pregnancy and lactation, it was shown that most of the studied women consumed some form of nutritional supplement or fortified food: 33.7% were supplemented with iron, 22.4% with multivitamins and minerals, 22.3% with iodine/iodized salt, 20.4% with folic acid and vitamin B12, and 32.7% with other supplements. A total of 26.2% even consumed milk enriched with calcium or other vitamins. In fact, only 25.2% of lactating mothers did not consume any type of supplement. On the other hand, Gila-Díaz et al. (2021) [2], in a study conducted on 30 healthy Spanish women during the lactation period (first month postpartum), observed that 57.1% of the women were taking dietary supplements during lactation.

In Poland, Bzikowska-Jura et al. (2021) [79] studied a group of 32 lactating mothers to assess their diets and evaluate its influence on human milk composition. Approximately half of the participants in this study (47%) declared consuming dietary supplements that included iron, and only 16% reported taking zinc supplements. Interestingly, it was observed that all the women who were supplemented with zinc also took iron supplements.

Although dietary supplement consumption is widespread in some European countries, only 5 out of 180 breastfeeding mothers in the ATLAS study cohort [64] reported taking dietary supplements. Likewise, in a study [63] involving 291 overweight women, where their diet was studied from pregnancy to the postpartum period (4 months), it was observed that only 24.4% of the women consumed dietary supplements during lactation, which was significantly lower than during the early pregnancy period.

In the United States, Jun et al. (2020) [107] performed a study to calculate the prevalence of supplement consumption in pregnant, lactating, and non-pregnant/non-lactating women and the contribution of micronutrients to the total intake in this group of women. This study was conducted between 1999 and 2014, using data from the National Health and Nutrition Examination Survey (NHANES). In a sample of 297 lactating women, it was observed that 70% were taking dietary supplements, of which 54% were taking prenatal supplements, which consisted mainly of multivitamins and minerals (at least three vitamins and one mineral). Additionally, in this study, 39.8% of lactating women indicated taking dietary supplements based on a healthcare professional’s recommendation, and 22.8% due to their own decisions. More than 60% of pregnant or lactating women were taking dietary supplements that contained thiamine, riboflavin, niacin, folic acid, vitamins B6, B12, C, and D, calcium, iron, selenium, and zinc. However, the prevalence of pregnant or lactating women taking other dietary supplements such as choline (5–8%), iodine (18–20%), magnesium (26–28%), phosphorus (5–6%), and selenium (10–12%) was low. Furthermore, it was observed that, due to the consumption of these dietary supplements, the majority of these women met the recommendations for thiamine, riboflavin, niacin, folic acid, vitamins B6, B12, and C, iron, and zinc. 

Another study [108] conducted in New Zealand showed that a high percentage of pregnant women included supplements in their diet: 96% of the sample included folic acid, 95% iodine, and 70% other dietary supplements (iron, calcium, magnesium, fish oil, selenium, zinc, vitamin C, B vitamins, probiotics, and vitamin D). However, during lactation, only 26% of these women continued to supplement with folic acid, and 63% with iodine.

Regarding vitamin D supplementation, Dawodu et al. (2015) [72], in a study conducted on lactating women (4 weeks postpartum) from China, the USA, and Mexico to examine sun exposure and vitamin D supplementation, reported a supplementation rate of 22% in lactating mothers from China, 94% in those from the USA, and 44% in those from Mexico. Moreover, Brembeck et al. (2016) [60] studied a group of 78 Swedish lactating women at 2 weeks, 4 months, and 12 months postpartum to observe their vitamin D statuses. This latter study found a reduction in vitamin D supplement intake as the first year of postpartum progressed—from 37% at 2 weeks postpartum and 31% at 4 months postpartum to 18% at 12 months postpartum. Moreover, only 8% of the sample consumed vitamin D supplements continuously during the first postpartum year.

As mentioned, there is still controversy regarding the necessity of supplementation during lactation. However, numerous research studies have observed beneficial effects following vitamin–mineral supplementation in lactating mothers, with a positive correlation found between the intake of these nutrients and their concentrations in human milk [109].

### 4.2. Influence of Vitamin Supplementation on Human Milk Composition

The effect of vitamin A or its various supplemental forms, including retinol, retinyl palmitate, red palm oil (rich in provitamin A), and β-carotene, on human milk content has been investigated. In some reviewed studies, vitamin A supplementation significantly raised retinol or β-carotene concentrations in human milk [110,111]. 

In a study where 435 lactating mothers were supplemented with 400,000 IU of vitamin A or a placebo, it was observed that while serum vitamin A levels were similar in both study groups, vitamin A supplementation was linked to significantly greater levels of retinol in human milk [112]. Significantly increased vitamin A levels in human milk were also observed after the administration of a single dose of vitamin A (60 mg) or placebo to a total of 9424 lactating mothers (in India, Peru, and Ghana) [113]. Finally, similar results were observed in other studies following a single oral dose of 209 μmol of retinol (200,000 IU of vitamin A) [114], after supplementation with a dose of retinyl palmitate (200,000 IU) [111,115,116,117,118], and after 209 nmol of retinol was administered [119].

In addition, positive effects on vitamin A concentrations in human milk were observed after supplementing a group of lactating mothers with 90 mg of β-carotene capsules and another group with the same amount but in the form of red palm oil. In this case, it was observed that the increase in β-carotene concentrations in human milk occurred in both groups, although this was significantly higher in the women who were administered red palm oil rich in this provitamin [110]. In another study by Canfield et al. (1999) [120], a group of 44 lactating vitamin A-deficient mothers was supplemented with 30 mg β-carotene capsules. After the intervention, a 9.6-fold increase in maternal serum β-carotene levels (0.092 ± 0.05 vs. 0.88 ± 0.38 μmol/L) and a 5.8-fold increase in human milk (0.017 ± 0.01 vs. 0.099 ± 0.09 μmol/L) were observed. On the other hand, Dijkhuizen et al. (2004) [121] found that breast milk β-carotene concentrations were higher in all women supplemented with β-carotene (4.5 mg/d), but breast milk retinol concentrations were higher only in women who received β-carotene + zinc. (4.5 mg β-carotene along with 30 mg of zinc daily). 

However, other studies found no association between vitamin A supplementation and the concentration of this vitamin in human milk. In an intervention study where high doses of vitamin A (in the form of retinyl palmitate) were administered to 197 lactating women deficient in this vitamin from Gambia, no vitamin concentration increase in human milk was observed [122]: no positive results were obtained after supplementation with 100–150% of the recommended vitamin A intake [123] or when administering 30 mg of β-carotene per day for 4 weeks [124]. In a study by Klevor et al. (34), the recommended intakes of this vitamin (800 retinol equivalents) were given to 1320 pregnant women, and the impact was evaluated (6 months postpartum): no increase was observed when compared to the control group [125]. Similarly, Tomiya et al. (2017) [126] administered 200,000 IU of retinyl palmitate in capsules, which also contained 40 mg of vitamin E (in one or two doses), and they did not find an increase in the concentrations of vitamin A in human milk in either of the two groups.

In general, results from randomized controlled trials that analyze the effects of maternal vitamin A supplementation postpartum showed a significant enhancement in maternal serum retinol, retinol in human milk, and hepatic vitamin A stores after a single dose of vitamin A supplements. It has been suggested that the baseline status of vitamin A in human milk could affect the outcomes of supplementation studies [127]. It appears that mothers who exclusively breastfeeding for a continued 6-month period showed a greater need for vitamin A supplementation compared to mothers who breastfed less frequently when practicing mixed feeding. Furthermore, as mentioned, comparing lactating mothers who start with very low concentrations of this vitamin at the beginning of lactation to mothers with normal or even high levels of this vitamin should be considered. However, in many of these studies, the technique used for human milk collection and whether the entire content of a feed was collected is not explained. Since vitamin A is fat-soluble and is carried in the lipid phase, the variability of milk fat content (time of day) should also be considered, which could lead to sampling errors due to non-standardized collection methods. This could explain the differences between the obtained results and does not allow for conclusive findings regarding the advisability of supplementing with vitamin A to increase vitamin A concentrations in human milk.

As for vitamin D, it is well known that during pregnancy maternal vitamin D status is closely coupled with fetal and neonatal vitamin D status. This connection continues during the lactation period [128]. Some studies show that maternal vitamin D status is better when the mother is exposed to sunlight, which improves the vitamin D fraction in human milk [129]. It is important to understand that human milk will be deficient in vitamin D when the mother has a deficiency in this vitamin [130]. Human milk usually contains a small amount of vitamin D [131,132,133], which has led to the common practice of supplementing breastfeeding infants (400 IU/day) [134]. However, several studies have shown that maternal supplementation with vitamin D could address both maternal and infant deficiency [135,136,137]. In fact, this recommendation, as an alternative to infant supplementation, is supported by the fact that daily administration of vitamin D by parents to breastfeeding infants is low in many regions of the world [138]. In the United States, reports of vitamin D supplementation rates in breastfeeding infants range from 9% to 20%, making infants reliant on the vitamin D content in human milk, which is usually deficient [139,140]. við Streym et al. (2016) [77] showed that exclusively breastfed infants received <20% of the daily dose (400 IU/d) recommended by the Institute of Medicine for infants during the first year of life [132]. Therefore, maternal supplementation of vitamin D can be a viable alternative to infant supplementation, safely addressing concerns for both the mother and the infant.

There are numerous studies linking vitamin D supplementation during lactation with increased levels of 25-hydroxy-vitamin D in human milk [141,142,143,144]. In a study by Ketha et al. (2018) [143], a group of breastfeeding mothers was supplemented with a single dose of 150,000 IU of vitamin D, while another group received a daily dose of 5000 IU of vitamin D for 28 days. The obtained results showed that the daily dose of 5000 IU was more effective in promoting the production of 1,25-dihydroxy-vitamin D. In contrast, the single high dose of vitamin D favored the production of 25,24-dihydroxy-vitamin D, with very limited physiological activity. Similar results were observed by Oberhelman et al. (2013) [142] in a group of breastfeeding mothers who received a daily dose of 5000 IU of cholecalciferol, where serum levels and concentrations in human milk significantly increased. In the group that received the single dose, an initial increase in vitamin D concentrations in maternal serum and human milk was quickly followed by a decrease. Moreover, Wall et al. (2016) [144] supplemented a group of pregnant women with a daily dose of 1000 IU of vitamin D3 and another group with two daily doses of vitamin D3 (2000 IU). They observed that the vitamin D concentrations in human milk were higher in the group that had been supplemented with 2000 IU of vitamin D3 during pregnancy. Other authors [145] supplemented a group of pregnant women during their third trimester with 1800 IU of vitamin D for 6 weeks, and they found that 25-hydroxy-vitamin D levels in the human milk of the vitamin D-supplemented group were significantly greater than in the placebo group.

It is important to remember that the vitamin D content of human milk is highly variable and can be influenced by seasons, maternal vitamin D intake, and even the ethnic background of the mother. Most studies have shown that maternal vitamin D supplementation increases the vitamin D content of human milk. However, it is also worth noting certain limitations in these studies. In the reviewed literature, the content of this nutrient was determined in human milk produced at different times postpartum (days, months, or years), which could have affected the studies’ results. Additionally, there appears to be a potential correlation between the intake of certain minerals (such as magnesium and zinc) and vitamin D levels [146,147,148].

It is also assumed that vitamin D toxicity is associated with hypercalciuria, hypercalcemia, and the risk of kidney stones [149]. The exact amount of vitamin D required to induce toxicity, i.e., the amount ingested over a specific period of time, is unknown in humans [150]. Vieth et al. [151] suggested, some years ago, that this amount would be 20,000 IU/day (500 μg/day). Hypervitaminosis D is a severe but very rare condition, and it has never occurred, to the best of our knowledge, when physiological amounts of this vitamin are consumed.

Hollis et al. (2015) [152] conducted a study to delve deeper into the effect of vitamin D supplementation in lactating mothers compared to supplementation in infants. In this study, it was observed that maternal supplementation with 6400 IU/day of vitamin D was safe and significantly increased blood vitamin D levels. Even when comparing the vitamin D statuses of infants receiving supplements (400 IU/day) with those of infants who did not receive supplements, no significant differences were observed. This means that maternal supplementation is adequate in meeting the vitamin D needs of the infant through human milk. This same study, as well as previous studies [136,137], did not report any toxicity with high doses of vitamin D (4000–6400 IU/day) in pregnant women. 

As for vitamin K, the literature reviewed [153,154,155] shows that supplementing lactating mothers with 2.5–5 mg of phylloquinone per day also increases the concentration of vitamin K (phylloquinone) in human milk. Similarly, in the case of vitamin E, studies [156,157,158] show that the levels of α-tocopherol in human milk increase after supplementation with a single dose of 400 IU of α-tocopherol. This increase in vitamin E concentration was observed in both colostrum and transitional milk but not in mature milk. This could be explained by increased fatty acid synthesis by the mammary gland in the first few days after birth due to the role of vitamin E in lipid metabolism. In fact, studies on the effect of maternal vitamin E supplementation on human milk are limited and inconclusive. Some limitations of previous studies include the small number of subjects they included, and some studies suggest that a single megadose of 400 IU of vitamin E may not be sufficient to increase the vitamin E content in human milk for an extended period [156].

Regarding dietary supplementation with B-group vitamins, supplementing with vitamins B1, B2 [159], B6 [160], and B12 [161,162] was associated with higher levels of these vitamins in human milk. However, as mentioned earlier, some studies observed that maternal supplementation with thiamine increased the content of this vitamin in human milk. Nevertheless, other studies did not observe any effect of thiamine supplements on the human milk of well-nourished women [163,164]. Supplementation with riboflavin was indeed associated with higher levels in human milk [165,166]. 

On the other hand, in a study by Chang et al. (2002) [160], a group of 47 breastfeeding mothers was supplemented with different doses of vitamin B6 (2.5, 4, 7.5, or 19 mg/day), and it was observed that the higher the dose of supplementation, the higher the concentration of this vitamin in human milk. In addition, Duggan et al. (2014) [162] supplemented 183 women during pregnancy and lactation with 50 mg of vitamin B12, and at 6 weeks postpartum it was observed that the concentration of this vitamin in human milk was significantly higher than in the placebo group. In another study [161], a group of 68 women was supplemented with 250 μg/day of vitamin B12, 60 mg/day of iron, and 400 μg/day of folic acid during pregnancy and the first 3 months postpartum. The supplementation of vitamin B12 during pregnancy and lactation resulted in increased concentrations of this vitamin in maternal plasma, colostrum, and human milk. However, in a study by Hampel et al. (2017) [167], a multivitamin supplement containing thiamine, riboflavin, and vitamins B6, B12, A, and E was administered, and a substantial increase in vitamin B6 concentrations in human milk was observed, but no significant changes were noted for thiamine, vitamin B12, vitamin A, and vitamin E.

For breastfeeding women who follow a vegan diet, as this type of diet carries a high risk of nutritional deficiencies in many nutrients, with vitamin B12 being one of the most crucial, supplementation with this vitamin is essential [168,169]. In a study by Pawlak et al. (2018) [168] which evaluated the use of B12 supplements in lactating vegan, vegetarian, and non-vegetarian American mothers and their impact on human milk, it was observed that 78.4% of the whole sample consumed supplements containing vitamin B12. The doses varied between 4 and 5000 µg, exceeding the recommendations for lactating mothers in all cases. It is worth noting that, in this study, there were significant differences in the percentage of participants using supplements that exclusively provided B12: 44.8% of vegan breastfeeding mothers, 26.2% of vegetarian breastfeeding mothers, and only 3.9% of non-vegetarian breastfeeding mothers. Pawlak et al. (2018) concluded that no differences were observed in terms of the content of vitamin B12 in human milk among the different groups of mothers studied, because mothers following plant-based diets and taking vitamin B12 supplements had concentrations in human milk comparable to those of mothers consuming diets that included animal products. However, it was observed that 20% of the sample, regardless of their diet type, had a lower level of vitamin B12 in human milk than the suggested thresholds for adequate infant intake.

As for folic acid, in a recent systematic review [170] folic acid supplements consumed during lactation were also directly associated with red blood cell folate levels. They could even be positively associated with serum folate. However, there was not enough available evidence to establish the relationship between the folic acid in dietary supplements consumed during lactation and hemoglobin concentrations, mean corpuscular volume, and serum vitamin B12.

Regarding vitamin C, in a study conducted by Daneel-Otterbech et al. (2005) [171], it was observed that supplementation with 1000 mg of vitamin C for ten days in lactating mothers significantly increased its concentration in human milk. However, other authors [172] did not find significant changes after the administration of vitamin C supplements (90 mg on the first day, followed by higher concentrations of 250, 500, or 1000 mg/day of vitamin C). Not much information is available on the impact of increased vitamin C intake on breastmilk. Additionally, a higher dose of vitamin C (1000 mg ascorbic acid/d for 5 d) resulted in a smaller response (in terms of vitamin C content in human milk) than lower doses (100 mg ascorbic acid/d for 10 d) administered over a longer period [171]. Furthermore, the differences in study results suggest significant deviation in the ascorbic acid content of human milk among individuals living in different geographic regions, depending on the amount of milk expressed in each sample and the analytical technique used.

### 4.3. Influence of Mineral Supplementation on Human Milk Composition

Regarding iron, most studies concur that iron supplementation does not significantly affect human milk concentrations of this mineral [173,174,175]. Breymann et al. (2007) [173] showed that the administration of just one intravenous dose of 100 mg of iron sucrose in 10 lactating women (with functional iron deficiency) at 2–3 days postpartum did not result in any changes in comparison to its baseline concentrations in human milk. Meanwhile, Holm et al. (2017) found no significant differences when comparing the effects of a single intravenous dose (1200 mg) of iron isomaltose to a daily oral iron dose (70.5 mg) in 65 lactating women after one week postpartum [176]. Similarly, Yalcin et al. (2009) [175] administered 80 mg of ferrous sulphate to 47 healthy women and concluded that iron supplement administration to non-anemic lactating women did not alter the iron content in human milk. Very similar results were obtained by Zapata et al. (1994) [174], who observed that the administration of iron sulphate tablets containing 40 mg of iron to 28 lactating mothers (aged 18–35) did not significantly modify the concentrations of this mineral in human milk. Nevertheless, supplementation with this mineral increased the total iron-binding ligands in human milk, determined by the total iron-binding capacity of lactoferrin in total secreted protein. Thus, lactoferrin levels in human milk appeared to be higher in women taking dietary supplements.

On the other hand, selenium supplementation was observed to increase levels in human milk in most of the available studies. For instance, Dodge et al. (1998) [177] administered 50 μg per day (as selenomethionine) or a placebo to 22 healthy young women aged 20–30 years during pregnancy and the first three months postpartum, resulting in a significant selenium increase in human milk. However, glutathione peroxidase enzyme activity remained unchanged. Interestingly, selenium supplementation also increased PUFA levels in human milk, especially those of linoleic acid, and decreased the concentration of SFAs. In another study, 20 μg/day of sodium selenate was administered to 23 lactating women, and a significant increase in selenium concentrations in human milk was observed at 3 and 6 months postpartum [178]. Similar results were reported by Moore et al. (2000) [179] after administering selenium supplements (100 µg as selenomethionine) to 21 Chinese women (a country historically known for low selenium intake) during pregnancy. In this case, selenium levels in human milk were higher in the supplemented group of women, although no differences in glutathione peroxidase enzyme activity were found between the two groups of women. Likewise, in another study [180], 200 μg of selenium per day in the form of yeast and sodium selenate was administered to 67 lactating women aged 19–39 years. After one month of supplementation, selenium concentration in human milk was significantly higher. However, Flax et al. (2014) [181] found that supplementation with sodium selenite (75 µg) was not related to changes in selenium concentrations in plasma or human milk.

Concerning zinc supplementation, in a study where women were supplemented daily with 15 mg (as ZnSO47H2O) during lactation, a significantly lower rate of decrease in zinc concentrations during lactation in the supplemented group compared to the control group was observed [182]. In another study conducted by Shaaban et al. (2005) [183], where 10 mg/day of zinc sulphate was administered to 60 lactating women, significantly higher concentrations in the human milk, hair, and nails (body stores) of lactating mothers were observed. On the other hand, Han et al. (2022) [184] studied a group of lactating mothers from Singapore and New Zealand to observe how the consumption of dietary supplements containing zinc (10 mg/day as zinc glycinate chelate) affected its concentration in human milk. The sample was divided into an intervention group (supplement including zinc) and a control group (supplement without zinc). It was observed that the zinc concentration in human milk during the first 3 months of lactation was 11% higher in the intervention group compared to the control group. However, in another study [185] where 20 mg of zinc sulphate, 2 mg of copper sulphate, and 116 mg of potassium iodide were administered to 32 women during lactation, a significantly decreased concentration of zinc in human milk was found during the study period in all lactating mothers. There were no significant differences in the rate of decrease in the concentration of this mineral between the women who started supplementation during lactation and those who received no dietary supplements. To summarize, somewhat contradictory results have been reported, which makes it difficult at present to establish a recommendation regarding supplementation.

Regarding iodine supplementation, the World Health Organization currently recommends the consumption of iodized salt as the best method to ensure adequate iodine intake throughout the population. In countries where the majority of households do not consume iodized salt, women should take a daily supplement to guarantee recommended iodine intakes [186]. Although in many countries, iodized salt consumption is usually sufficient to meet iodine intake recommendations in children and adults, it has been observed to remain insufficient in lactating mothers [187,188]. Therefore, the American Thyroid Association advises that lactating women should receive a 150 µg iodine supplement daily [189]. The use of daily iodine supplements to address deficiency has limitations since there are various barriers to their use, and absorption is often suboptimal [190]. In Norway, a study [86] was conducted in 175 lactating mothers, where they observed that only 29% of the sample regularly took a supplement (150–200 µg) containing iodine. In those mothers who consumed iodine supplements, significantly higher levels of iodine were observed in human milk compared to in mothers who did (157 µg/L vs. 72 µg/L). Considering that, as mentioned earlier, the iodine content in human milk is directly dependent on the mother’s intake, and as breastfeeding is recommended for at least the first 6 months, proper iodine consumption in the mother is crucial, and supplementation, if necessary, is essential. In a study [191] conducted on Swedish lactating mothers, it was observed that even though most women had a slightly inadequate iodine intake, only 15% of the sample used supplements containing iodine. The iodine content in these supplements varied widely, ranging from 10 to 240 µg/day. Additionally, iodine concentrations in human milk were higher in women who consumed iodine supplements. Another study [85] involving 206 postpartum mothers showed that 24% of the sample used dietary supplements, but only 10% used supplements containing iodine, with an average iodine intake provided by these supplements of 110 µg/day. In New Zealand, Jin et al. (2022) [87] conducted a study with 87 lactating women and their children to examine the iodine statuses of both groups during the postpartum period (3, 6, and 12 months). They observed that 44% of the sample was consuming iodine-containing supplements (150–250 μg iodine/day). However, they noticed that the consumption of iodine-rich supplements decreased to only 6% of the sample at 12 months postpartum. In only the first 3 months of the postpartum period did women who included iodine supplements have higher levels of iodine in their milk compared to those who did not use iodine-rich supplements.

Excess iodine consumption can lead to thyrotoxicosis and hypothyroidism. There are no studies showing adverse effects for the lactating mother or her child when women consume iodized salt or iodine-containing oils [192]. In fact, in developing countries where there is a high prevalence of iodine deficiency, supplementation with iodized oil prevented children from hypothyroidism for 3 years, also reducing their risk of mortality [193]. 

Regarding calcium supplementation during breastfeeding, studies indicate that calcium supplements do not have a significant effect on increasing the calcium content in human milk (1–1.5 g calcium carbonate/d) [194,195,196]. However, other studies [197,198] show a benefit for the mother (since this is a time when her demands for calcium greatly increase due to the loss through human milk), suggesting that higher calcium intake during early lactation could decrease bone loss in mothers with a daily calcium intake below recommendations. 

Of interest, while studies have evaluated supplementation with individual micronutrients in lactating mothers, very few have studied supplementation with multiple micronutrients. This highlights the need for more studies to evaluate the effectiveness and safety of nutritional supplements in lactating women as well as their infants in order to obtain more conclusive results.

### 4.4. Influence of Omega-3 Polyunsaturated Fatty Acid Supplementation on Human Milk Composition and Maternal Health

The role of long-chain PUFAs in myelination, central nervous system development, and visual development during the perinatal period is now well established. The accumulation of DHA in the brain occurs rapidly during the second half of gestation and the first year after birth, suggesting that this is a critical period for adequate supply from the diet, adipose stores, or synthesis from precursor fatty acids (ALA) [199].

The effects of prenatal omega-3 fatty acid supplements on a child’s neurocognitive development likely depend on the mother’s dietary intake of omega-3 fatty acids as well as the child’s ability to produce sufficient amounts of these long-chain PUFAs from their own precursor fatty acids [200]. In developed countries, supplementation during lactation has mainly focused on supplementing with long-chain PUFAs. Studies have shown that supplementation with omega-3 (*n*-3) fatty acids increases the level of DHA in human milk [45] and is associated with improved neonatal health outcomes [12]. 

In most of the reviewed research studies [104], where dietary supplements of omega-3 fatty acids were administered during pregnancy (200–2200 mg/day of DHA and 100–1100 mg/day of EPA for approximately 20 weeks), it was observed that supplementation improved some parameters indicative of infant cognitive development. It is true that for some of these parameters, inconsistent or insufficient evidence was obtained in relation to other aspects of a child’s neurocognitive development. Additionally, populations with lower socioeconomic status were underrepresented, and the studies lacked racial and ethnic diversity.

Jensen et al. (2005) [201] conducted a randomized controlled trial in which they supplemented a group of breastfeeding mothers with DHA derived from algae oil (200 mg DHA/day) and another group with a placebo (vegetable oil) during the first 4 months postpartum to observe how the increased DHA consumption in lactating mothers affected the neurodevelopment and visual function of their babies. They observed that DHA supplementation resulted in higher DHA content in maternal plasma (50%), human milk (75%), and children’s blood plasma (35%). Additionally, the children of mothers supplemented with DHA scored higher on a test assessing psychomotor development (Bayley Psychomotor Development Index) compared to children in the control group. In a subsequent study (2010) [202], children from the previous study were re-evaluated after 5 years. They found that 5-year-old children whose mothers received DHA supplements during the first 4 months of breastfeeding performed better on a sustained attention test (Leiter International Performance Scale) compared to those who received the placebo. This, along with the improved performance reported in the earlier study, suggests that DHA intake during early childhood provides long-term benefits in different aspects of neurodevelopment. 

However, a systematic review [203] carried out by Cochrane in 2015 stated that there were “no conclusive findings to support or refute the practice of administering long-chain PUFA supplements to breastfeeding mothers to improve neurodevelopment or visual acuity”. Another review [204] conducted in 2016 reached the same conclusion. Even a more recent systematic review [104] in 2021 concluded that omega-3 supplementation during pregnancy may be beneficial for infants’ cognitive performance. Nevertheless, the evidence was not strong enough to evaluate the effects of omega-3 PUFA supplements during pregnancy and/or lactation on language development, physical or motor development, academic performance, or the risks of attention-deficit disorder, attention-deficit/hyperactivity disorder, autism spectrum disorder, anxiety, or depression.

On another note, a recently conducted meta-analysis of randomized trials (2022) [205] stated that there was moderate evidence for the positive effect of omega-3 PUFA supplementation in pregnant and lactating women. This suggests that omega-3 supplementation during pregnancy may have favorable effects against preeclampsia, low birth weight, preterm birth, and postpartum depression, and may improve infant growth (anthropometric measurements), immune system development, and visual activity.

## 5. Conclusions

It is worth noting that women during lactation tend to have a lower-quality dietary pattern when compared to pregnancy, even though their nutrient requirements are higher for the most part. Moreover, there is also a marked decrease in dietary supplement consumption during lactation.

In general, a very positive association has been observed between nutritional supplementation in lactating mothers and nutrient concentration in human milk. Vitamin and/or mineral supplements that contain fat-soluble vitamins (A, D, E, and K), B-group vitamins (B1, B2, B6, B12), and vitamin C, as well as some minerals (selenium, zinc, iodine, and magnesium) are the ones that have the greatest effect on the composition of human milk. In addition, very positive results have also been obtained with supplementation of polyunsaturated omega-3 fatty acids. 

Given the limitations found in the reviewed literature (significant individual variation in dietary intake, potential overestimation and/or underestimation of intake, differences in analytical techniques used, and methods of human milk sample collection), the need for intervention studies that assess the effectiveness and safety of nutritional supplements in lactating women, as well as their infants, should be emphasized in order to obtain more conclusive results and inform subsequent public health recommendations.

## Figures and Tables

**Table 1:** International fat intake recommendations and evidence of compliance with nutritional recommendations.

	Recommendations		Evidence of Compliance with Nutritional Recommendations	
	EFSA (2010) [17]	WHO/FAO (2010) [18]	Below the Recommendations (*n*, Studies)	Meet/Above the Recommendations (*n*, Studies)
Lipids (% TE)	20–35	20–35	*n* = 0	*n* = 1 [19]
SFA (% TE)	ALAP	10	*n* = 0	*n* = 9 [1,19,20,21,22,23,24,25,26]
MUFA (% TE)	-	By difference ^a, b^	*n* = 2 [23,25]	*n* = 4 [1,20,21,26]
PUFA (% TE)	-	6–11	*n* = 1 [1]	*n* = 2 [20,25]
LA *n*-6 (% TE)	4 *	2.5–92–3 *	*n* = 0	*n* = 3 [1,19,26]
*n*-3 (% TE)	-	0.5–2	*n* = 3 [27,28,29]	*n* = 0
ALA (% TE)	0.5 *	0.5	*n* = 1 [27]	*n* = 3 [1,19,29]
DHA mg/day	350–450 *	200 (EPA + DHA = 300)	*n* = 8 [1,19,22,23,26,27,28,29]	*n* = 0

ALA: alpha-linolenic acid; SFA: saturated fatty acids; MUFA: monounsaturated fatty acids; PUFA: polyunsaturated fatty acids; LA: linoleic acid; DHA: docosahexaenoic acid; EPA: eicosapentaenoic acid; ALAP: as low as possible; % TE: % Total Energy. EFSA: European Food Safety Authority; WHO/FAO: World Health Organization/Food and Agriculture Organization. * AI: Adequate intake. ^a^ Total fat [%E]—SFA [%E]—PUFA [%E]—TFA [%E]. ^b^ Can be up to 15–20% E, according to total fat intake.

**Table 2:** International fat-soluble vitamin intake recommendations for breastfeeding mothers and evidence of compliance with nutritional recommendations.

	Organization Recommendations		Evidence of Compliance with Nutritional Recommendations	
	EFSA (2017) [57]	WHO/FAO (2005) [58]	Below the Recommendations (*n*, Studies)	Meet/Above the Recommendations (*n*, Studies)
Reference Intakes	AI	NIR		
Vitamin A ^b^ (μg/day)	1300 ^a^	850 ^c^	*n* = 7 [1,2,59,60,61,62,63]	*n* = 1 [59]
Vitamin D (μg/day)	15	5	*n* = 8 [1,2,59,60,61,62,64,65]	*n* = 0
Vitamin E ^d^ (mg/day)	11	-	*n* = 0	*n* = 2 [2,59]
Vitamin K (μg/day)	70	55	-	-

^a^ EFSA values for vitamin A are PRI (population reference intake) instead of AI. ^b^ RE: Retinol Equivalents (1 μg RE is equivalent to 1 μg of retinol, 6 μg of β-carotene, and 12 μg of other provitamin A carotenoids). ^c^ WHO/FAO values for vitamin A are “recommended safe intakes” instead of NIR. ^d^ Expressed as mg of α-tocopherol per day. AI: adequate intake; NIR: nutritional intake of reference. (-) not available. EFSA: European Food Safety Authority; WHO/FAO: World Health Organization/Food and Agriculture Organization.

**Table 3:** International water-soluble vitamin intake recommendations for breastfeeding mothers and evidence of compliance with nutritional recommendations.

	Organization Recommendations		Evidence of Compliance with Nutritional Recommendations	
	EFSA (2017) [57]	WHO/FAO (2005) [58]	Below the Recommendations (*n*, Studies)	Meet/Above the Recommendations (*n*, Studies)
Vitamin B1 (mg/day)	1.1	1.5	*n* = 3 [60,61,63]	*n* = 4 [2,59,62,64]
Vitamin B2 (mg/day)	2 ^a^	1.6	*n* = 1 [63]	*n* = 4 [1,2,59,62]
Vitamin B3 (mg/day)	16 ^b^	17 ^b^	*n* = 2 [61,63]	*n* = 3 [1,2,64]
Vitamin B5 (mg/day)	7 ^c^	7	-	-
Vitamin B6 (mg/day)	1.7 ^d^	2	*n* = 3 [60,61,63]	*n* = 4 [1,2,59,62]
Folic Acid (μg/day)	500 ^e, f^	500	*n* = 5 [1,2,59,60,63]	*n* = 0
Vitamin B12 (μg/day)	5	2.8	*n* = 1 [61]	*n* = 3 [2,6,62]
Biotin (μg/day)	45	35	-	-
Vitamin C (mg/day)	155 ^g^	70	*n* = 2 [63,64]	*n* = 2 [1,59]

^a^ EFSA values for vitamin B2 or riboflavin are PRI/AI. ^b^ Values for vitamin B3 or niacin are expressed in mg of NE (1 mg of niacin = 1 niacin equivalent or NE = 60 mg of tryptophan). ^c^ EFSA values for vitamin B5 or pantothenic acid are AI. ^d^ EFSA values for vitamin B6 or pyridoxine are PRI/AI. ^e^ EFSA values for vitamin B9 or folate are PRI/AI. ^f^ EFSA and WHO/FAO values for vitamin B9 or folate are expressed as mg of dietary folate equivalents or DFE. DFE can be calculated as μg DFE = μg of food folate + (1.7 × μg of folic acid). ^g^ EFSA values for vitamin C are PRI. AI: adequate intake; NIR: nutritional intake of reference; EFSA: European Food Safety Authority; WHO/FAO: World Health Organization/Food and Agriculture Organization.

**Table 4:** International mineral recommendations for breastfeeding mothers and evidence of compliance with nutritional recommendations.

	Organization Recommendations				Evidence of Compliance with Nutritional Recommendations	
	EFSA (2017) [57]	WHO/FAO (2005) [58]			Below the Recommendations (*n*, Studies)	Meet/Above the Recommendations (*n*, Studies)
Reference Intakes	AI	NIR				
Calcium (mg/day)	975	1000 ^a,b,c^			*n* = 4 [1,59,61,79]	*n* = 5 [2,19,64,80,81]
Magnesium (mg/day)	300	270 ^a,b,c^			*n* = 1 [20]	*n* = 3 [59,64,79]
		High bioavailability	Moderate bioavailability	Low bioavailability		
Zinc (mg/day)	12.2	5.8 ^a^	9.5 ^a^	19 ^a^	*n* = 4 [59,64,79,82]	*n* = 4 [1,27,61,81]
		5.3 ^b^	8.8 ^b^	17.5 ^b^		
		4.3 ^c^	7.2 ^c^	14.4 ^c^		
Sodium (mg/day)	2000	-			*n* = 1 [1]	*n* = 5 [59,64,79,80,83]
Potassium (mg/day)	4000	-			*n* = 5 [1,2,59,64,79]	*n* = 1 [80]
Phosphorous (mg/day)	550	-			*n* = 0	*n* = 5 [1,20,59,79,81]
Iron (mg/day)	16	10–30 ^a,b,c,d^			*n* = 6 [20,27,59,79,81,84]	*n* = 4 [2,61,64,80]
Iodine (μg/day)	200	200 ^a,b,c^			*n* = 6 [2,59,64,79,85,86]	*n* = 2 [2,87]

^a^ 0–3 months, ^b^ 3–6 months, ^c^ 7–12 months, ^d^ according to bioavailability. AI: adequate intake; NIR: nutritional intake of reference. (-) not available. EFSA, European Food Safety Authority; WHO/FAO, World Health Organization/Food and Agriculture Organization.

## Data Availability

Not applicable.
